# Establishing a common instantaneous center of rotation for the metatarso-phalangeal and metatarso-sesamoid joints: a theoretical geometric model based on specific morphometrics

**DOI:** 10.1186/s13018-019-1110-4

**Published:** 2019-04-16

**Authors:** Michael Durrant, Lara Durrant, Tucker McElroy

**Affiliations:** 1 Borrego Community Health Foundation, Borrego Springs, CA 92004 USA; 2 Loma Linda University, Loma Linda, CA 92354 USA; 3 Center for Statistical Research and Methodology, U.S. Census Bureau, Washington, D.C., USA

**Keywords:** Fibular sesamoid, Foot kinematics, Functional morphology, Geometric model, Instantaneous center of rotation, Metatarsal motion, Sesamoid, Sesamoid groove, Tibial sesamoid

## Abstract

**Background:**

Previous research has identified separate sagittal plane instantaneous centers of rotation for the metatarso-phalangeal and metatarso-sesamoid joints, but surprisingly, it does not appear that any have integrated the distinctive morphological characteristics of all three joints and their respective axes into a model that collectively unifies their functional motions. Since all joint motion is defined by its centers of rotation, establishing this in a complicated multi-dimensional structure such as the metatarso-phalangeal-sesamoid joint complex is fundamental to understanding its functionality and subsequent structural failures such as hallux abducto valgus and hallux rigidus.

**Methods:**

Based on a hypothesis that it is possible to develop an instantaneous center of rotation common to all four osseous structures, specific morphometrics were selected from a sequential series of 0.5-mm sagittal plane C-T sections in one representative cadaver specimen randomly selected from a cohort of nine, seven which were obtained from the Body Donation Program, Department of Anatomy, University of California, San Diego School of Medicine, and two which were in the possession of one author (MD). All mature skeletal specimens appeared grossly normal, shared similar morphological features, and displayed no evidence of prior trauma, deformity, or surgery. Specific C-T sections isolated the sagittal plane characteristics of the inter-sesamoidal ridge and each sesamoid groove, and criteria for establishing theoretical sesamoid contact points were established. From these data, a geometric model was developed which, to be accurate, had to closely mimic all physical and spatial characteristics specific to each bone, account for individual variations and pathological states, and be consistent with previously established metatarso-phalangeal joint functional motion.

**Results:**

Sequential sagittal plane C-T sections dissected the metatarsal head from medial to lateral and, at approximately midway through the metatarsal head, the circular nature of the inter-sesamoidal ridge (crista) was isolated; other C-T sections defined, respectively, the elliptical characteristics of the tibial (medial) and fibular (lateral) sesamoid grooves in each specimen. A general plane model representing the most basic form of the joint was developed, and its center of rotation was established with a series of tangential and normal lines. Simplified tibial sesamoid and fibular plane models were developed next which, when combined, permitted the development of a spherical model with three separate contact points. Based on the morphometrics of each sesamoid groove and a more distally positioned tibial sesamoid, the model was modified to accurately define the center of rotation and one distinctive sagittal plane geometric and functional characteristic of each groove.

**Conclusion:**

Consistent with our hypothesis, this theoretical geometric model illustrates how it is possible to define an instantaneous center of rotation common to all three joints while simultaneously accounting for morphometric and spatial variability. This should provide additional insight into metatarso-phalangeal-sesamoid joint complex functionality and the physical characteristics that contribute to its failure.

## Introduction

Due to the complexity of the metatarso-phalangeal-sesamoid joint complex (MPSJC), there are few studies that have attempted to establish its instantaneous centers of rotation (ICR); we are only aware of the following five [1–5]. Defining and integrating specific morphometrics unique to each bone and their articular surfaces is essential to determining its ICR. Any geometric model developed must have the capability of defining MPSJC motion in three planes while simultaneously permitting analysis of different sesamoid spatial arrangements and allowing for variant morphometric anomalies that may adversely affect its stability. To avoid more complex vector analysis, the model presented here is simplified by aligning the metatarsal, proximal phalanx, and both sesamoids in the sagittal plane, with the understanding that it must also be capable of simultaneously integrating other planes of motion seamlessly. A different geometric perspective of the MPSJC has been previously presented by Yoshioka and colleagues [6]; five metatarsophalangeal joint cadaver specimens were embedded in resin which were sequentially sectioned in the sagittal plane. They established the congruent symmetrical relationships between each groove and its respective sesamoid and noted both grooves were oriented to the long axis of the metatarsal.

## Methods

**Fig. 1:**
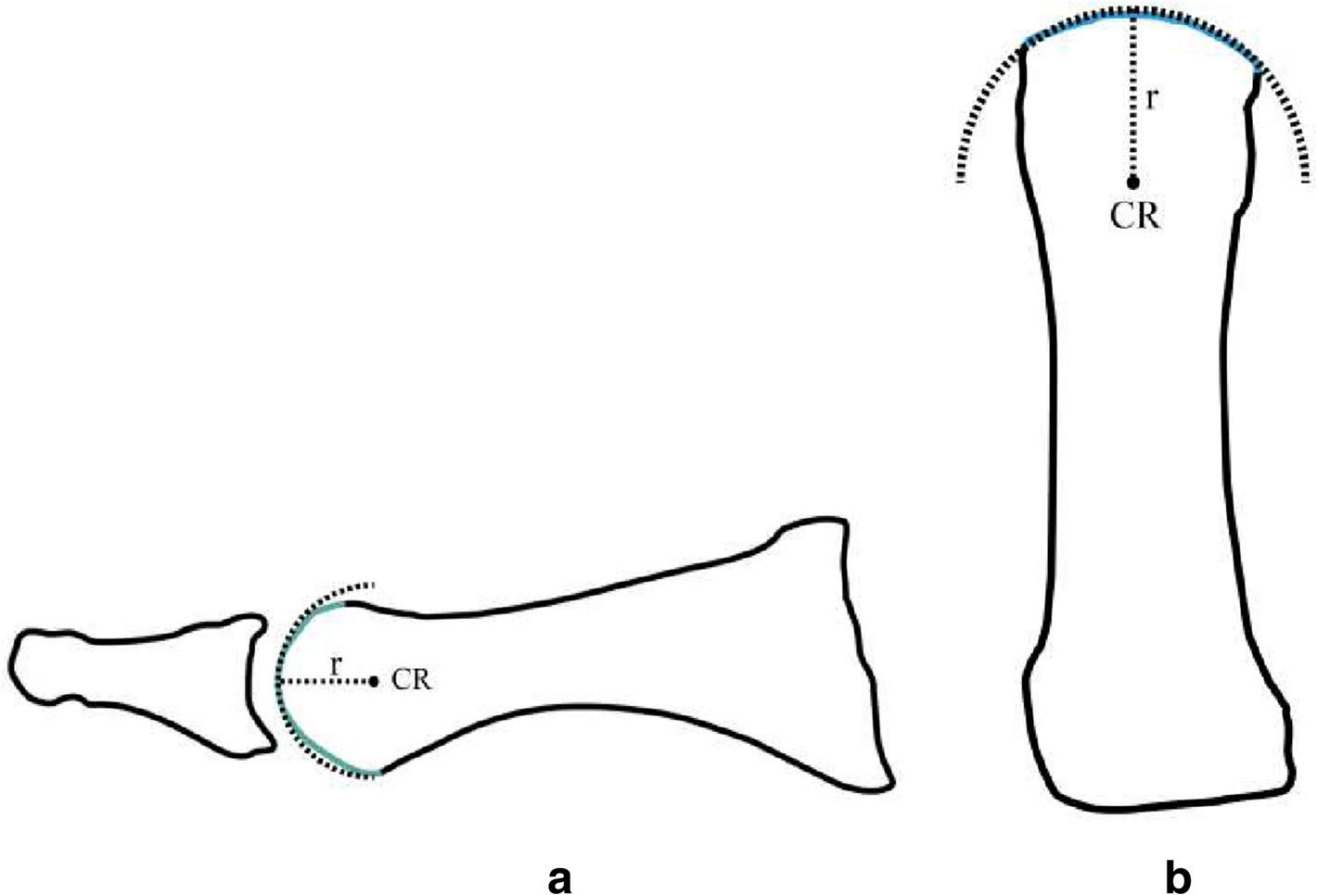
A sagittal plane shadow drawing of C-T section 23, midway through the metatarsal specimen, defining its intersesamoidal ridge (crista), is illustrated in Figure **a**. Its distal cartilaginous surface, highlighted in teal, has been described as a convex spherical surface ([7, 4, 8, 9, 6, 10]). Figure **b** is a shadow drawing of the same metatarsal specimen from a dorsal perspective, highlighting its circular form in the transverse plane. A black dotted semi- circle (first described by Stokes and colleagues [11]), with its center of rotation designated CR, has been superimposed over the distal metatarsal edge in the sagittal and transverse planes to illustrate its distal spherical surface. The length of radius in figure 1b is slightly longer than 1a, which results in its distal surface being more flattened in the transverse plane than the sagittal plane figure in 1a. Although both images exhibit two geometrically different convex circular surfaces, it is possible to integrate both CR into one functional model

A sagittal cross section of the metatarsal head through its inter-sesamoidal ridge, also known as the crista (C-T section 23), in Fig. 1a, reveals a circular metatarsal head, which has been described as a convex spherical surface [4, 6–10].

**Fig. 2:**
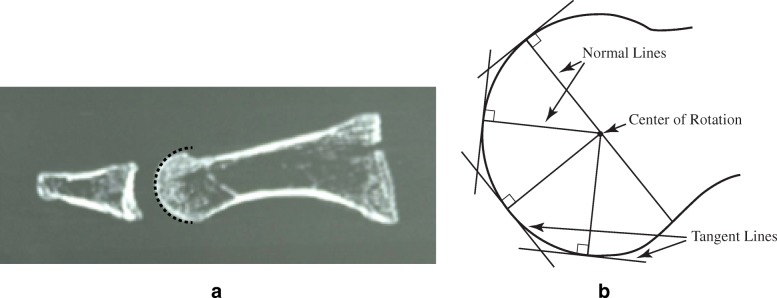
C-T section 23 in **a** is the approximate midpoint through the metatarsal head, at the inter-sesamoidal ridge in our specimen. **b** illustrates a general circular plane model denoting the distal C-T section 23 in **a**. Note that normal lines drawn perpendicular to tangential lines define its borders and help establish a CR. These normal lines, due to the geometry of the circle, will always intersect at the circle’s center

A circle in the sagittal plane with two points on its perimeter is first considered; tangent lines to these points can be constructed, and their normal lines are similarly defined as inward pointing lines perpendicular to the respective tangent. These normal lines, due to the geometry of the circle, will always intersect at the circle’s center. Illustrated in Fig. 2b, this approach to determining a CR is also valid for more elliptical surfaces. By definition, elliptical is any curved surface which is less than that of a sphere of comparable size (i.e., the elliptical surface is more flat than a sphere in certain portions, similar to the ellipsoid of analytic geometry).

**Fig. 3:**
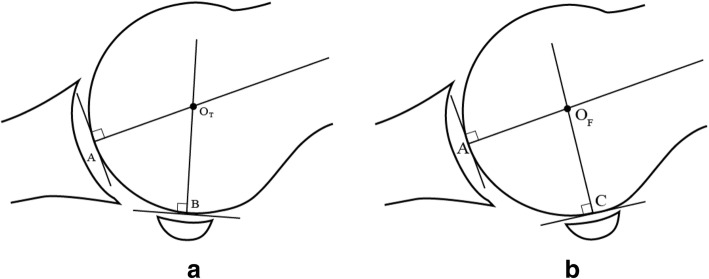
**a** illustrates a tibial plane model, with normal lines drawn perpendicular to tangential lines intersecting contact points for the tibial sesamoid and proximal phalanx. Their point of intersection determines its CR. **b** illustrates the fibular plane model, with specific contact points for the proximal phalanx and fibular sesamoid. Again, the intersecting normal lines define its CR. Since the fibular sesamoid contact point is more proximal, point C is also positioned more proximal (relative to the distal metatarsal edge) than point B in **a**

Next, consider a sagittal cross section, known as the tibial plane, with two contact points, A designating the contact point between the metatarsal and proximal phalanx, and B, representing the contact point between the metatarsal and tibial sesamoid. This idealized tibial plane is determined by A, B, and the requirement that the cross section resembles a spherical metatarsal head. In this simplified model, it is possible to establish a CR by locating the intersection of the normal lines of A and B, as illustrated in Fig. 3a. This CR, designated OT_,_ defines distal metatarsal motion.

It is also possible to model the fibular plane, which is determined by contact points A and C (with the understanding that its cross section also be circular), where C is the contact point between the metatarsal and the fibular sesamoid. It is similarly possible to establish a CR, which is designated by OF (see Fig. 3b). Consistent with previous observations [4, 11–16] that position the fibular sesamoid more proximal, in comparing Fig. 3a and b, notice that the fibular sesamoid contact point at C is positioned more proximal relative to tibial sesamoid contact point B.

It is fairly easy to determine the coordinates of *OT* and *OF* with respect to the points A and B and the points A and C. These models give two separate CR—one for the tibial plane, representing rotation about an axis perpendicular to the tibial plane, and one for the fibular plane, which represents rotation about an axis perpendicular to the fibular plane. While each of these models is less complicated than a three-dimensional model, they can account for two CR.

**Fig. 4:**
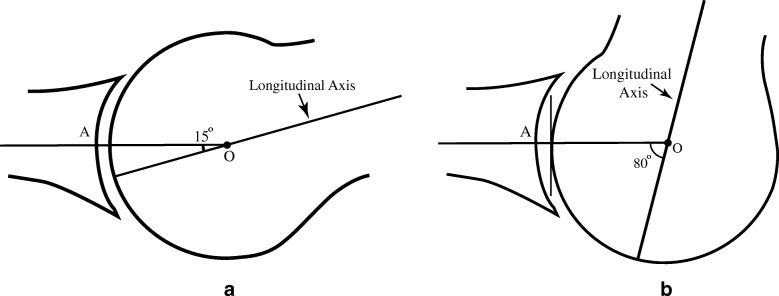
**a** represents an initial 15° metatarsal declination angle, and **b** illustrates its approximate terminal declination angle

It is necessary for this model to account for multiple metatarsal declination angles that define its sagittal plane position at any instant of time. These angles are referenced to a stationary proximal phalangeal base, designated as A. This angle is determined by finding the angle subtended between the normal line from A and the longitudinal axis of the metatarsal. This angular relationship between the two bones helps determine the initial direction and variable amount of compressive force projected toward the metatarsal from distal during stance and propulsive gait. Multiple authors have established an initial metatarsal declination angle range of 12–20° [17–22]. This initial declination is designated *θ*0 and is illustrated in Fig. 4a; the CR is designated O.

The final angle, when the metatarsal has reached its highest declination angle, is designated *θ*M and illustrated in Fig. 4b. This declination angle is uniquely variable with each individual and has been determined to be 65–75° [4, 17, 19, 22–26]. In between the initial angle *θ*0 and *θ*T, there are multiple declination angles that describe the metatarsals precise position at every second from static stance to end stage propulsion and toe off.

## Results

**Fig. 5:**
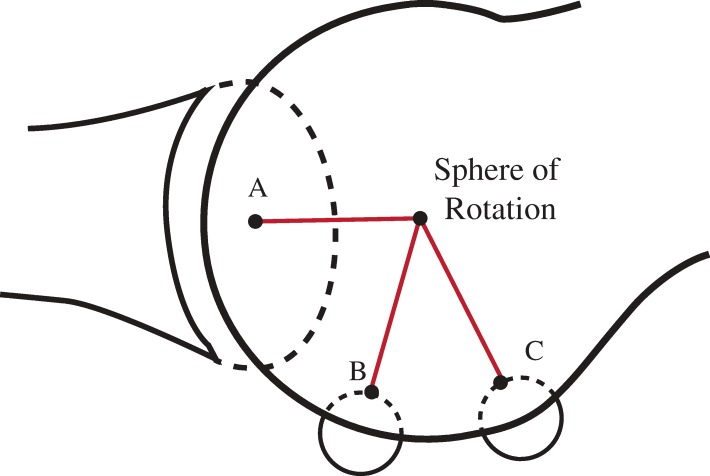
illustrates that, by establishing precise contact points and combining the intersecting normal lines from the proximal phalanx and each sesamoid, it is possible to construct a three-dimensional model with an instantaneous sphere of rotation

The two axes of rotation that were established for the tibial and fibular planes in Fig. 3a and b can now be integrated, and their point of intersection is defined as their shared ICR (Fig. 5). This sphere of rotation is dictated by the geometry of the distal metatarsal surface, which may shift at any second as it rotates. Obviously, if the metatarsal head is perfectly spherical, this point will not change, but when the curvature of the metatarsal head is altered (i.e., its plantar elliptical grooves), it is possible for the instantaneous sphere of rotation to simultaneously shift in any direction. Frick [18], and somewhat later Steinler [27] both observed three planes of motion in the metatarso-phalangeal joint which require three separate axes. Other researchers have also noted the capability of a shallow ball and socket joint to similarly facilitate multi-planes of motion simultaneously [4, 8, 28, 29] (Fig. 5).

This model is consistent with observations that have described the sesamoids and proximal phalanx as a single anatomical and functional apparatus (platform) over which the metatarsal head rotates [7, 13, 30, 31] and is its base of support. Although yet to be determined in the MPSJC, research has demonstrated that it is possible to define precise contact points between adjacent bones [32–34]. This model is constructed in a manner supportive of a position that the metatarsal, with very small exceptions, is the only structure in the MPSJC that exhibits measurable motion during propulsion [35, 36]. And because all closed chain metatarsal motion is passive [18, 27], it is possible to define its motion primarily by forces projected to its cartilaginous surface by both sesamoids and proximal phalanx. As constructed, this theoretical model will also accommodate randomly selected contact positions and, by nature of its ICR capacity, is capable of accommodating variable sesamoid sizes and spatial arrangements, different metatarsal and phalangeal lengths and declination angles, and multiple planes of motion.

## Discussion

**Fig. 6:**
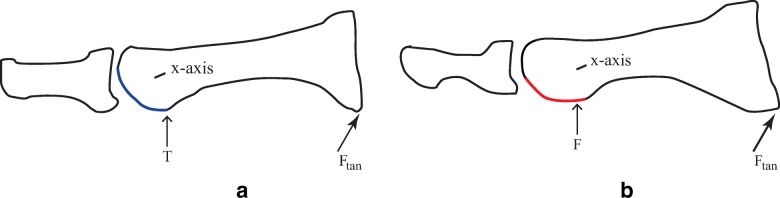
**a** is a shadow drawing of C-T section 31 representing the sagittal section defining the approximate trough of the tibial groove. Note its elliptical (flattened) surface, which almost immediately starts to curve somewhat perpendicular to the metatarsal longitudinal axis. **b** is a shadow drawing representing C-T section 15 defining the approximate trough of the fibular groove. The fibular groove begins more proximal, with its proximal flattened surface more parallel to the longitudinal axis of the metatarsal. Tibial sesamoid contact point at the proximal aspect of its groove is designated T; the initial fibular sesamoid contact point in its groove is designated F. Placing a Ftan at the base of the metatarsal illustrates how the different elliptical characteristics of each groove will affect its CR and motion distal and proximal to its x-axis

The basic principles of this geometric model can be expanded to incorporate the uniquely different elliptical characteristics of each tibial and fibular groove, which were isolated by select sagittal C-T sections of this skeletal specimen. The approximate trough of each groove was isolated and determined to be C-T section 31 for the tibial groove and CT section 15 for the fibular groove; their shadow drawings are illustrated in Fig. 6a and b.

To simplify the models in Fig. 6a and b, it is necessary to select a contact point which is constant and can be consistently defined in every individual; the proximal inception point of each sesamoid groove was used as a reference, designated T and F in Fig. 6a and b.

**Fig. 7:**
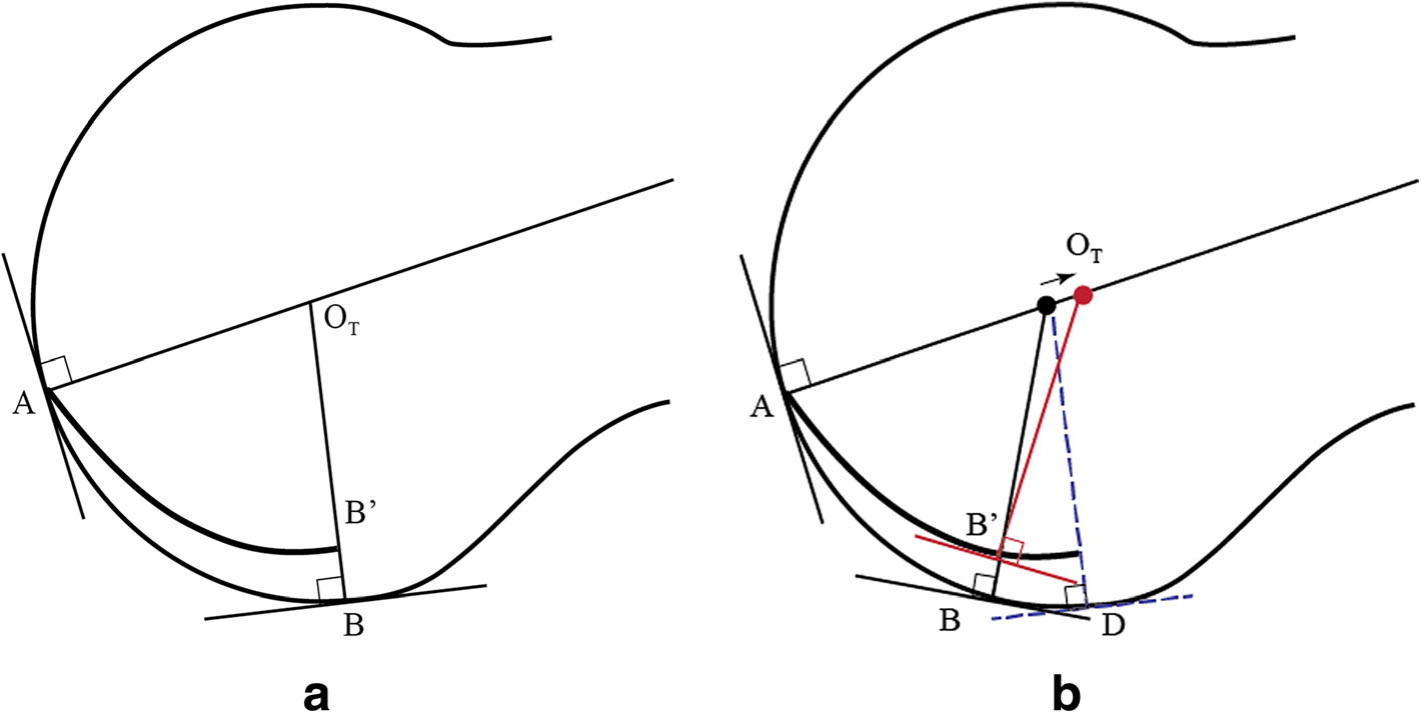
**a** and **b** illustrate initial and subsequent tibial sesamoid positions on the ridge and groove. **a** represents the initial tibial sesamoid contact point in its groove (B’), whereas B would be its position if the metatarsal were spherical. In **b**, it can be seen that there is a change in the CR (red normal line) due to the groove having a different tangent line (B’) from the ridge (B); it has shifted proximal because the elliptical tibial groove makes the tangent lines of A and B’ more obtuse than the angle between the tangent lines at A and B, and as a result, it has shifted proximal in **b**

Defining the distal surface of the metatarsal by a series of tangent lines that follow the metatarsal head and tibial and fibular grooves rather than the inter-sesamoidal ridge, and utilizing normal lines, the intersection of points A and B (focusing on the tibial contact point) respectively will initially begin and end in the same position as does the spherical model of the metatarsal head (see Fig. 7a, which gives a depiction of the tibial contact point). Figure 7a shows that there is no difference in the CR between a model in which the sesamoid is on the ridge or in its groove, since the tangent lines are parallel at both contact points B and B’. But in Fig. 7b, these tangent lines are no longer parallel; when the sesamoid contact point is positioned further distal in its groove, its CR is actually more proximal than in the former case. This demonstrates the effect of groove curvature on the location of the CR. Figure 7a depicts the tibial contact point, but the fibular contact point is similar in principle, although it is actually positioned more proximal.

**Fig. 8:**
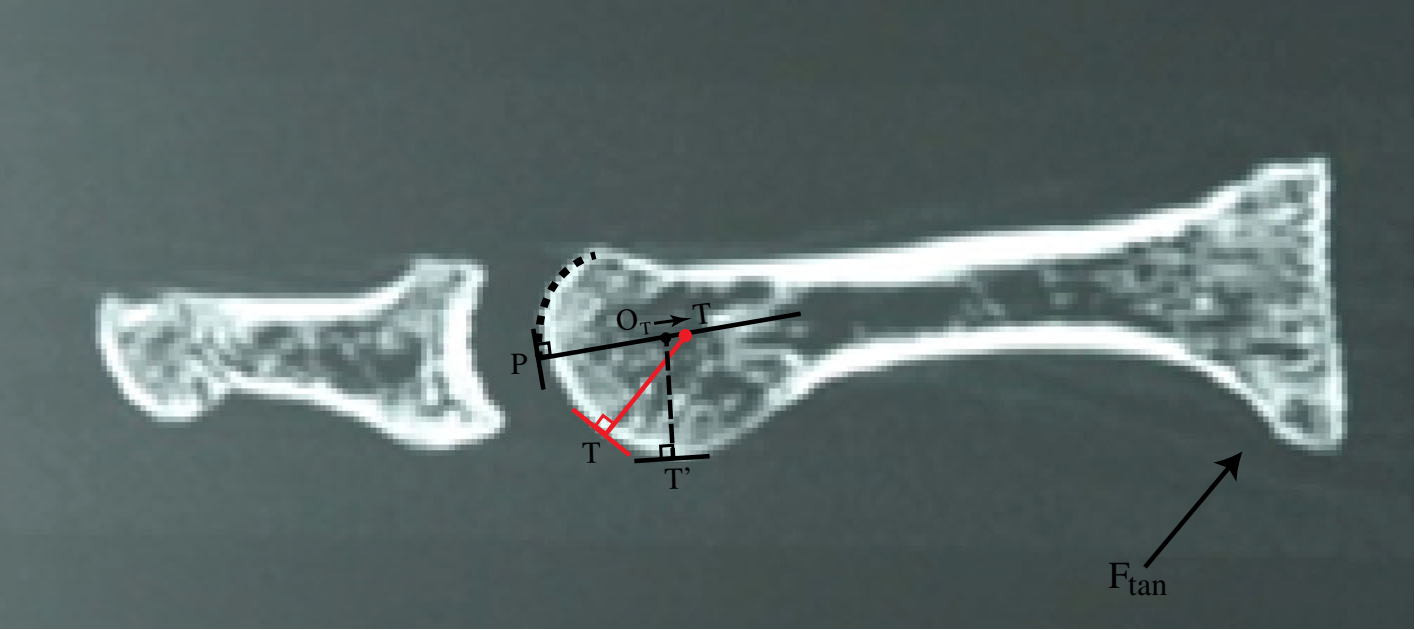
is the enlarged sagittal C-T section 31 illustrating the approximate tibial groove trough in shadow drawing 31 in Fig. 6a. We have taken the concept illustrated in Fig. 7a and b and applied it to this image. An assumption is made that the tibial groove commences immediately, where its elliptical surface changes direction. The dotted black line defines the partial circular shape of the distal dorsal metatarsal edge. The CR will shift proximal in response to the geometry of the tibial groove rather than the phalanx

Figure 7b demonstrates how the normal line at B’ is significantly altered from the normal line at B. There must exist a range of declination angles for which the tangent lines at B and B’ are no longer parallel; the angle subtended between the tangent lines of A and B’ is more obtuse than the angle between the tangent lines at A and B. The result of this difference between the tangent lines at B and B’ is that the normal line at B’ is pointed more proximal than the normal line at B. Since the normal line at B’ is initially pointed in the same direction as B’s normal line, there must be a shift in this normal line as the tibial groove rotates over the tibial sesamoid. Assuming that the normal line at contact point A is fixed (which depends on the implicit sphericity of the metatarsal head), the tibial CR must shift proximal. Of note, near the metatarsal’s highest declination angle, the contact points B and B’ will once again be identical, since groove depth will have completely diminished by then. This requires the medial CR to shift distal, close to its original position. (Fig. 8).

While the anatomical shape and curvature of the metatarsal are not as precise as in our geometric model, we have chosen to model it in this more simplistic form in order to determine its CR. Models are simplified inaccurate pictures of reality, but are useful to the extent that they capture the structure’s salient features in a tractable form. The models in Fig. 7a and b gives a simplified view of the tibial plane, and illustrates how its CR will initially shift proximal, and then will shift distal later. However, since the fibular sesamoid is located (when referenced to the distal metatarsal edge) proximal to the tibial sesamoid, it follows that *OT* will shift proximal prior (in the course of propulsion) to the shift of *OF*.

**Fig. 9:**
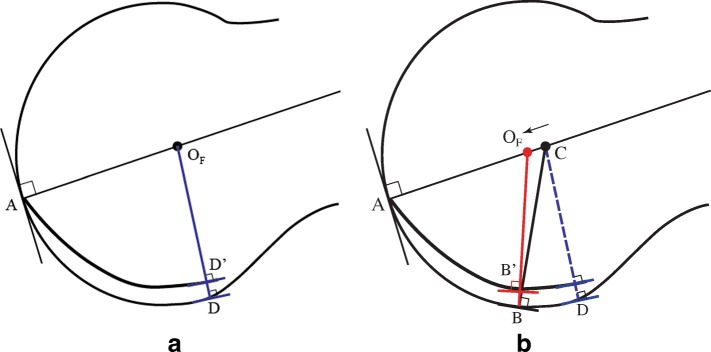
**a**, the fibular sesamoid contact point has been placed at the proximal end of its groove. **b**, it can be seen that there is a change in the CR due to the groove having a different tangent line (B′) from the ridge (B). It has shifted distal in **b**

**Fig. 10:**
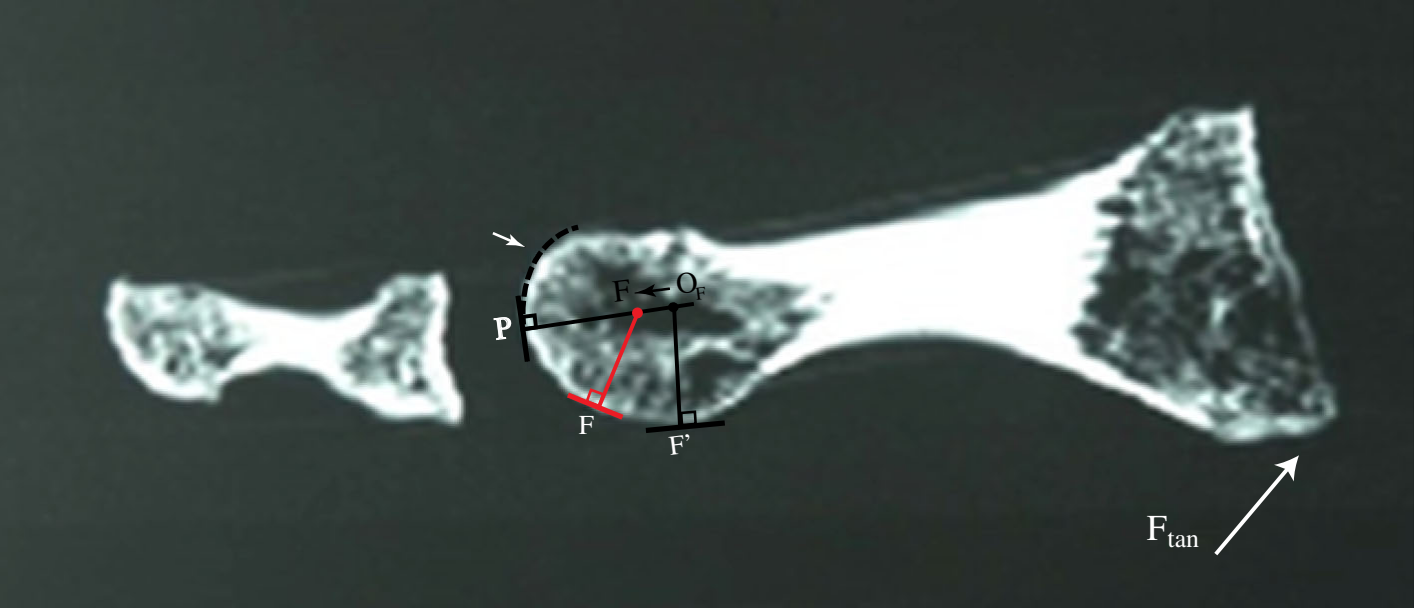
is the C-T section defining the approximate trough of the fibular groove; its shadow drawing 15 is visible in Fig. 6b. We have taken the concept illustrated in Fig. 9a and b and applied it to this image; note that the CR has shifted distal. An assumption is made that the fibular groove commences immediately, where its proximal flat surface begins. Similar to the tibial shadow section, the white arrow delineates the partial circular morphometric shape of the distal dorsal aspect of the metatarsal

When Fig. 9b is examined, it can be easily seen that the flattened portion of the fibular groove begins at its proximal inception point, so that the elliptical portion of its groove appears to be more parallel to the metatarsal longitudinal axis. It is here at the distal portion of its groove that the normal line at B’ is significantly altered from the normal line at B. Again, there must exist a range of metatarsal declination angles for which the tangent lines at B and B’ are no longer parallel; the angle subtended between the tangent lines of A and B’ is now more acute than the angle between the tangent lines at A and B (see Fig. 9b). The result of this difference between the tangent lines at B and B’ is that the normal line at B’ is pointed more distal than the normal line at B. Keeping in mind that the normal line at B’ is initially pointed in the same direction as B’s normal line, this implies a shift in the normal line as the fibular groove rotates over its sesamoid. Assuming that the normal line at contact point A is fixed (which depends on the assumed sphericity of the metatarsal head), the fibular center of rotation must shift distal. Furthermore, near the highest metatarsal declination angle, contact points B and B’ will be identical because groove depth will have diminished by this point; this will result in the fibular CR again shifting proximal, close to its original position (Fig. 10).

Figure 7a and b give a simplified view of the tibial plane and illustrate that the tibial CR will shift proximal. However, since the fibular sesamoid is positioned more proximal (Figures 9a and 9b) than the tibial sesamoid, OT will shift prior to the shift of OF.

There are multiple benefits to a distal shift in the sesamoids CR—it better aligns with the intrinsic plantar tendons and sesamoids at higher metatarsal declination angles. It significantly reduces the angular and rotational surface velocities of the distal metatarsal edge, while increasing them slightly at its proximal end. And importantly, it increases the amount of force required to displace the metatarsal head dorsally, thereby diminishing the prospect of a metatarsus elevatus from occurring.

There is substantial evidence that certain aberrant morphological characteristics and spatial arrangements increase the risk of hallux limitus (HL), hallux rigidus (HR), and hallux abducto valgus (HAV), and for this model to be credible, its ICR must be capable of responding to abnormalities. A flattened distal metatarsal articular surface increases the potential for linear plane deformities such as HL and HR by keeping both the phalanx and metatarsal functionally aligned in the sagittal plane [37]. Hetherington et al. [4] observed 2° of metatarsal abduction as the metatarsal, distal to its *x*-axis, plantarflexed; the ICR in this model would be able to accommodate this transverse plane motion.  Proximally positioned sesamoids [5, 14–16, 38, 39] have been implicated in HL and HR deformities; these spatial positions will result in increased compressive shear forces between their dorsal surfaces and the metatarsal grooves at higher declination angles. Restricting end range metatarsal plantarflexion distal to its *x*-axis (see Fig. 6a, b) can ultimately lead to jamming and degenerative joint disease associated with HR. The relationship between elongated metatarsals and HL and HR has been known since metatarso-phalangeal joint disorders were first investigated [23, 40–46]. If the sesamoids are positioned more proximal, relative to the distal metatarsal edge, then the ICR must also be positioned more proximal.

First noted by Inge and Ferguson [47], and validated by subsequent studies, the tibial sesamoid is normally positioned more distal [15, 16, 39, 48, 49]. Siebel [50] first noted that this spatial relationship will place the *x*-axis slightly oblique to the longitudinal axis of the metatarsal; this will result in its ICR being positioned slightly more lateral proximal. Importantly, the obliquity of this *x*-axis helps facilitate metatarsal inversion around its longitudinal axis, which has been shown to be critical for first ray stability [8, 51–53]. Any condition that initially or subsequently places the metatarsal in an everted position increases the likelihood that its functional inversion motion will be incomplete which, over time, increases the risk for ligamentous strain, hypermobility, and HAV. Factors contributing to this frontal plane deformity include surgical excision of the tibial sesamoid [54, 55], its congenital absence [56, 57], high metaphyseal eversion torsion [58], first metatarsal pronation [59–64], and a loss in the medial longitudinal arch [60]; all scenarios place the metatarsal in an excessively everted position. A failure to surgically address high metatarsal eversion angles can result in recurrent HAV [53, 65–69]. Common to these disorders is that they all collectively position the ICR more medial plantar; each can easily be assimilated into this geometric model. A short first metatarsal has been implicated in HAV [70, 71]; because of diminished ground reactive forces (GRF), there will be inadequate forces to drive metatarsal inversion, which will result in a lack of first ray stability. In this case, the ICR will exhibit very little normal frontal or transverse plane motion. A rounded distal metatarsal edge (and diminished length of radius) has been implicated in HAV [72], which females are predisposed to [73]; the ICR will be positioned more distal resulting in more potential frontal plane motion and instability. It is also possible for this model to accommodate multiple sesamoid sizes and shapes, including hypertrophied sesamoids, all of which have also been implicated in HL, HR, and HAV [14, 39].

## Conclusion

Consistent with our hypothesis, this analysis demonstrates that it is possible to assimilate specific morphometric characteristics of each bone and their spatial relationships into a geometric model of the MPSJC with a common ICR. Modifications to reflect other specific morphological features, and establishing all three axes of motion in each individual bone, including defining its ICR, are all prerequisites for precisely establishing metatarsal position at each declination angle during gait. An analysis of GRF passing through each sesamoid and the phalanx prior to being projected to specific contact points on the distal and plantar aspect of the metatarsal head should further help define its motion. Finally, it seems obvious that a more complete understanding of MSPJC functionality and the underlying etiologies of HR and HAV will require a more comprehensive orthogonal coordinating system and an expanded morphometric list to more accurately define its multiple planes of motion and ICR.

## Abbreviations

CR: Center of rotation
C-T: Computed tomography
GRF: Ground reactive forces
HAV: Hallux abducto valgus
HL: Hallux limitus
HR: Hallux rigidus
ICR: Instantaneous center of rotation
MPSJC: Metatarso-phalangeal-sesamoid joint complex
OF: Fibular plane center of rotation
OT: Tibial plane center of rotation
UCSD: University of California, San Diego
